# Interstitial deletion of the *Apc* locus in β-catenin-overexpressing cells is a signature of radiation-induced intestinal tumors in C3B6F1 *Apc^Min^*^/+^ mice^†^

**DOI:** 10.1093/jrr/rrad021

**Published:** 2023-04-28

**Authors:** Hiromi Yanagihara, Takamitsu Morioka, Shunsuke Yamazaki, Yutaka Yamada, Hirotaka Tachibana, Kazuhiro Daino, Chizuru Tsuruoka, Yoshiko Amasaki, Mutsumi Kaminishi, Tatsuhiko Imaoka, Shizuko Kakinuma

**Affiliations:** Department of Radiation Effects Research, National Institute of Radiological Sciences, National Institutes for Quantum Science and Technology, Chiba, Japan; Department of Radiation Effects Research, National Institute of Radiological Sciences, National Institutes for Quantum Science and Technology, Chiba, Japan; Department of Radiation Effects Research, National Institute of Radiological Sciences, National Institutes for Quantum Science and Technology, Chiba, Japan; Department of Radiation Effects Research, National Institute of Radiological Sciences, National Institutes for Quantum Science and Technology, Chiba, Japan; Department of Radiation Effects Research, National Institute of Radiological Sciences, National Institutes for Quantum Science and Technology, Chiba, Japan; Department of Biology, Graduate School of Science, Chiba University, Chiba, Japan; Department of Radiation Effects Research, National Institute of Radiological Sciences, National Institutes for Quantum Science and Technology, Chiba, Japan; Department of Biology, Graduate School of Science, Chiba University, Chiba, Japan; Department of Radiation Effects Research, National Institute of Radiological Sciences, National Institutes for Quantum Science and Technology, Chiba, Japan; Department of Radiation Effects Research, National Institute of Radiological Sciences, National Institutes for Quantum Science and Technology, Chiba, Japan; Department of Radiation Effects Research, National Institute of Radiological Sciences, National Institutes for Quantum Science and Technology, Chiba, Japan; Department of Radiation Effects Research, National Institute of Radiological Sciences, National Institutes for Quantum Science and Technology, Chiba, Japan; Department of Radiation Effects Research, National Institute of Radiological Sciences, National Institutes for Quantum Science and Technology, Chiba, Japan

**Keywords:** *Apc^Min^* ^/+^ mouse, immunoguided laser microdissection, interstitial deletion, intestinal tumor, radiation signature

## Abstract

Recent studies have identified interstitial deletions in the cancer genome as a radiation-related mutational signature, although most of them do not fall on cancer driver genes. Pioneering studies in the field have indicated the presence of loss of heterozygosity (LOH) spanning *Apc* in a subset of sporadic and radiation-induced intestinal tumors of *Apc^Min^*^/+^ mice, albeit with a substantial subset in which LOH was not detected; whether copy number losses accompany such LOH has also been unclear. Herein, we analyzed intestinal tumors of C3B6F1 *Apc^Min^*^/+^ mice that were either left untreated or irradiated with 2 Gy of γ-rays. We observed intratumor mosaicism with respect to the nuclear/cytoplasmic accumulation of immunohistochemically detectable β-catenin, which is a hallmark of *Apc*^+^ allele loss. An immunoguided laser microdissection approach enabled the detection of LOH involving the *Apc*^+^ allele in β-catenin-overexpressing cells; in contrast, the LOH was not observed in the non-overexpressing cells. With this improvement, LOH involving *Apc*^+^ was detected in all 22 tumors analyzed, in contrast to what has been reported previously. The use of a formalin-free fixative facilitated the LOH and microarray-based DNA copy number analyses, enabling the classification of the aberrations as nondisjunction/mitotic recombination type or interstitial deletion type. Of note, the latter was observed only in radiation-induced tumors (nonirradiated, 0 of 8; irradiated, 11 of 14). Thus, an analysis considering intratumor heterogeneity identifies interstitial deletion involving the *Apc*^+^ allele as a causative radiation-related event in intestinal tumors of *Apc^Min^*^/+^ mice, providing an accurate approach for attributing individual tumors to radiation exposure.

## INTRODUCTION

Ionizing radiation is a carcinogen that induces cancer by causing DNA double-strand breaks with subsequent mis-rejoining of those breaks. It remains uncertain, however, whether cancer risk can be attributed to low radiation doses (or rates), for which the expected effect is small and below the level of statistical significance given the relatively high frequency of sporadic cancer [1]. To assess whether cancer risk can be attributed to low-level radiation exposure, biomarkers reflecting a key event in carcinogenesis would be of great use [2]. Recent cancer genome resequencing studies have indicated that candidate biomarkers include certain common interstitial deletions and balanced inversions observed in secondary cancers of patients who have received radiation therapy and thyroid cancers diagnosed in people who once resided near the Chernobyl nuclear accident site. Although those biomarkers are concordant with the aforementioned consequences of ionizing radiation, they do not overlap with the tumor suppressor genes that are commonly mutated in various cancers [3, 4]. The existence of such radiation-induced mutations in a causative gene would strongly suggest cancer causation by radiation and thus may be useful for estimating the risk of developing cancer after low dose exposure to radiation.

Epidemiological studies, including those of Japanese atomic bomb survivors, have revealed that radiation exposure increases the risk of developing stomach and colon cancers [5, 6], indicating that the gastrointestinal tract is highly susceptible to radiation-induced cancer. Colon cancer is one of the most common cancers worldwide, and molecular studies have demonstrated the involvement of accumulated mutations in multiple genes, including *APC* (adenomatous polyposis coli) [7]. *APC* inactivation leads to nuclear accumulation of β-catenin, which activates canonical Wnt signaling. β-catenin binds to the LEF/TCF family of transcription factors that activate genes promoting cell proliferation and survival, which may lead to adenomas; these precursor lesions oftentimes accumulate further genetic mutations that can lead to colon cancer. Germline mutations in *APC* cause familial adenomatous polyposis (FAP) [8]. Moreover, *Min* (multiple intestinal neoplasia) encodes a nonsense mutation of the murine *Apc* locus. *Apc^Min^*^/+^ mice harbor a germline *Apc* mutation (g.2549T>A, p.850L>X) associated with multiple intestinal neoplasms mainly in the small intestine, and thus, these mice are one of the most widely used models of FAP [9, 10].

The *Apc^Min^*^/+^ mice, if they have the pure genetic background of strain C57BL/6J (B6), develop large numbers of spontaneous intestinal polyps (mostly adenomas and their precursor lesions); owing to the severe hemorrhage associated with these polyps, B6 *Apc^Min^*^/+^ mice typically die within 20 weeks after birth [11]. Molecular analyses have revealed that the wild-type *Apc* allele (*Apc*^+^) is lost in spontaneous intestinal tumors of B6 *Apc^Min^*^/+^ mice [12]. On the other hand, *Apc^Min^*^/+^ mice on a F1 hybrid background of B6 and C3H strains (C3B6F1) develop much fewer polyps than B6 *Apc^Min^*^/+^ mice and hence live long enough for sufficient observation of progression from adenoma to adenocarcinoma [13]. In addition, with F1 *Apc^Min^*^/+^ mice, it is feasible to study the loss of heterozygosity (LOH) in intestinal tumors using polymorphic markers (e.g. microsatellite loci), and indeed, a radiation-specific LOH pattern involving the *Apc* locus has been found in tumors that developed after irradiation [14–16]. However, a subset of tumors in these studies retained the *Apc*^+^ allele [14–16], advocating the need for further clarification. The copy number variations associated with those LOH patterns also remain unresolved.

We have previously shown that exposure of male C3B6F1 *Apc^Min^*^/+^ mice to ionizing radiation increases the incidence of intestinal tumors [13]. Here, we report a combination of techniques applicable to this animal model, in which LOH and DNA copy number analyses were used to detect radiation-specific causative mutations as a measure for attributing the initiating event in individual tumors to radiation exposure. First, we needed to overcome the issue of intratumor heterogeneity inherent in the intestinal tumors of C3B6F1 *Apc^Min^*^/+^ mice. Second, by isolating β-catenin-overexpressing regions in the tumor, we identified a signature, namely interstitial deletions, that inactivates the *Apc*^+^ allele specifically in radiation-induced tumors.

## MATERIALS AND METHODS

### Mice and irradiation protocol

Male B6 *Apc^Min^*^/+^ mice were originally purchased from the Jackson Laboratory (Bar Harbor, ME, USA) and maintained in-house. Female C3H/HeJ mice were obtained from Charles River Laboratories (Kanagawa, Japan). Male B6 *Apc^Min^*^/+^ mice were intercrossed with female C3H/HeJ mice to obtain C3B6F1 *Apc^Min^*^/+^ hybrids, which were identified by genotyping as described [12]. The F1 mice were housed in a conventional clean room under the following conditions: 12-h dark/light cycle, temperature 23 ± 2°C and humidity 50 ± 10%. Animals were irradiated at 2 weeks of age and sacrificed at 30 weeks of age. Irradiation (γ-rays, 0.5 Gy/min) was performed using a Gammacell 40 irradiator (Nordion Inc, Ottawa, Canada) with a ^137^Cs source. X-ray irradiation (200 kVp, 0.6 Gy/min) was carried out using an X-ray generator (Pantak, East Haven, CT, USA) as described [17]. All animals were fed a laboratory rodent diet and given water *ad libitum*. All protocols of animal experiments were reviewed and approved by the Institutional Animal Care and Use Committee of the National Institutes for Quantum Science and Technology (approval number 14–1024 and 19–1005) and were performed in strict accordance with the Institutes of *Guide for Care and Use of Laboratory Animals*. All efforts were made to minimize suffering.

### Tumor isolation and fixation

Mice were sacrificed under isoflurane anesthesia, and the whole gastrointestinal tract was removed. The tract was then separated into stomach, small intestine, caecum and colon, with the small intestine divided further into three segments of equal length. The gut segments were cut along the line of the mesenteric attachment, spread onto paper strips with villi facing upward and fixed in 10% phosphate-buffered formalin for 24 h or in the PAXgene Tissue System fixative (Qiagen, Hilden, Germany) for 3 h, then stored in 70% ethanol. After recording the number and size of tumors larger than 0.5 mm in diameter (Leica Application Suite LAS V4.12; Leica Microsystems, Tokyo, Japan), the tumors were excised and embedded in paraffin.

### Histopathological examination and immunohistochemistry

Serial sections were cut at 5 μm thickness and stained with hematoxylin and eosin (H&E). Histological analysis was performed according to the previously reported criteria [18]. After dewaxing and hydration, sections were incubated with an antibody against β-catenin (dilution 1:200; clone 14, BD Biosciences, Franklin Lakes, NJ, USA), a peroxidase-conjugated secondary antibody [Histofine Simple Stain MAX PO (Mouse) kit, Nichirei, Tokyo, Japan], reacted with 3,3′-diaminobenzidine tetrahydrochloride and counterstained with hematoxylin.

### DNA extraction from tumor samples

Using a laser microdissection (LMD) system (MMI CellCut, Molecular Machines & Industries GmbH, Eching, Germany), tumor cells were microscopically collected from 50 to 100 sections in 0.5 ml tubes. DNA was subsequently prepared for polymerase chain reaction (PCR) using the QIAamp DNA Micro kit (Qiagen) with minor modifications. Briefly, tumor tissue was suspended in Buffer AL with proteinase K (Qiagen) and incubated at 56°C for 16 h. The DNA samples were heated at 90°C for 1 h, washed and eluted from the spin column prior to use.

### LOH analysis

LOH analysis of the *Apc* locus was performed using HiDi *Taq* DNA polymerase (myPOLS Biotec GmbH, Konstanz, Germany) and allele-specific primers (Table 1). The PCR cycling program was as follows: 95°C for 2 min and 57°C for 5 min, followed by 42 cycles of 72°C for 30 s, 95°C for 15 s and 57°C for 15 s, and finally 72°C for 2 min. LOH analysis of chromosome 18 microsatellite loci was carried out using Ampli*Taq* Gold polymerase (Thermo Fisher Scientific, Waltham, MA, USA). The PCR cycling program was 95°C for 9 min and 60°C for 5 min, followed by 20 cycles of 72°C for 15 s, 95°C for 15 s and 60°C for 15 s, 25 cycles of 72°C for 30 s, 95°C for 30 s and 60°C for 30 s, and finally 72°C for 7 min. Primers are listed in Table 1. The PCR products were analyzed by capillary electrophoresis (QIAxcel Advanced System, Qiagen).

**Table 1:** Primers for LOH analysis

Allele or locus	Forward primer (5′ to 3′)	Reverse primer (5′ to 3′)
*Apc* ^+^ allele	[F1]^a^ TTCTGAGAAAGACAGAAGTTT	[R1]^a^ TGTCGTCCTGGGAGGTATGA
*Apc^Min^* allele	[F2]^a^ TTCTGAGAAAGACAGAAGTTA	[R2]^a^ TTTGGCTATCTGGGCTGCAG
D18Mit64^b^	TCAGATTCACTGCTAAGTCTTTTC	AGCAAGAAAAGCAGGTGAGG
D18Mit119^b^	AGATGCTTGTGAAACATACATATGTG	GAGTGTATAGCGGACTTTTGGG
D18Mit120^b^	ACTGCACTGGTCCCATTTTC	CAATAGTTGGAAATCAGACAGGC
D18Mit124^b^	CCCAAATGGGGTGTCTTTTA	CTGCCACACATTTGTGTGTATG
D18Mit184^b^	CACACATGTGTAGGTAGGTAGGTAGG	CGCACAAGGACTACTGAAACA
D18Mit187^b^	TGCTTGAAGAAAGAGATCCTACG	GACATGCATGCCTGTAACTCC

^a^Primer [names (F, forward; R, reverse)].

^b^Microsatellite marker loci.

### Array comparative genomic hybridization

DNA samples were amplified using the Sigma-Aldrich GenomePlex WGA2 kit (Merck KGaA, Darmstadt, Germany). Whole-genome amplification (WGA) was carried out with 10–25 ng DNA. The amplified DNA was labeled with either cyanine 3 or 5 using the SureTag Complete DNA Labeling kit (Agilent Technologies, Santa Clara, MA, USA). The array comparative genomic hybridization (aCGH) analysis was performed as described [19, 20]. The microarray data have been deposited in the Gene Expression Omnibus database under accession number GSE228693.

### Statistical analysis

The data were analyzed using Prism 8.0 software (GraphPad Inc, San Diego, CA, USA). The statistical significance of differences between two sets of independent data was determined using the Student’s or Welch’s *t*-test based on the results of the *F*-test. The relative proportions of adenocarcinoma as well as LOH patterns were compared using Fisher’s exact test.

## RESULTS

### Radiation facilitates intestinal tumorigenesis in C3B6F1*Apc^Min^*^/+^mice

Mice were irradiated with 2 Gy of γ-rays at 2 weeks of age and sacrificed at 30 weeks of age. Mean body weight at the time of sacrifice was significantly lower in the irradiated male mice than the nonirradiated males (Table 2). Tumor incidence in the small intestine of C3B6F1 *Apc^Min^*^/+^ mice of both sexes was 100%; in the colon, irradiation slightly increased the incidence in males, but the difference was not significant (Table 2). Irradiation significantly increased the mean number of tumors in the small intestine of both sexes; in the colon, again, radiation nonsignificantly increased the number of tumors in males. Thus, radiation facilitated tumorigenesis in the small intestine of both male and female C3B6F1 *Apc^Min^*^/+^ mice.

**Table 2:** Frequency of intestinal tumors in C3B6F1 *Apc^Min^*^/+^ mice

Sex	Treatment	Mice	Body weight at sacrifice (g)^a^	Mice with tumors (%)^b^		Tumors/mouse^a^		Proportion of AC^c^ **(%)**	
				SI^d^	Colon	SI^e^	Colon	SI	Colon
Male	0 Gy	5	49.8 ± 3.2	5 (100)	2 (40)	15.4 ± 2.5	0.4 ± 0.5	14/63 (22)	1/2 (50)
	2 Gy	5	41.8 ± 2.6^**^	5 (100)	4 (80)	26.2 ± 9.1^*^	0.8 ± 0.4	29/87 (33)^*^	3/4 (75)
Female	0 Gy	5	38.8 ± 2.7	5 (100)	1 (20)	13.6 ± 3.8	0.2 ± 0.4	9/58 (16)	0/1 (0)
	2 Gy	5	36.8 ± 3.5	5 (100)	1 (20)	24.4 ± 4.1^**^	0.2 ± 0.4	22/73 (30)	0/1 (0)

^a^Data represent the mean ± SD.

^b^Includes adenoma and adenocarcinoma (AC).

^c^Only tumors >1.5 mm in diameter were subjected to pathology analysis using HE-stained sections.

^d^SI = small intestine.

^e^Number of tumors in each mouse (male, 0 Gy: 15, 19, 16, 15 and 12; male, 2 Gy: 29, 33, 33, 11 and 25; female, 0 Gy: 11, 14, 12, 11 and 20; female, 2 Gy: 23, 19, 29, 28 and 23).

^*^ *P* < 0.05.

^**^ *P* < 0.005 vs 0 Gy.

### Radiation increases the incidence of adenocarcinoma among intestinal tumors

**Fig. 1:**
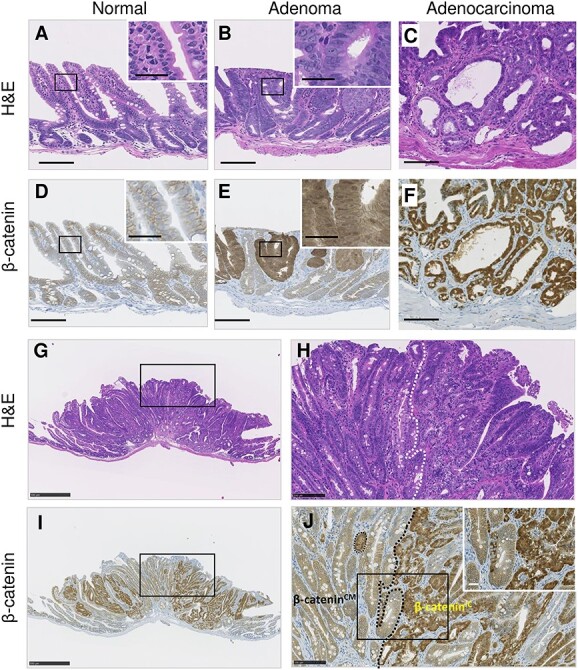
Representative histology and β-catenin immunostaining images of normal and neoplastic intestinal FFPE tissues. (**A**–**C**, **G** and **H**) H&E staining. (**D**–**F**, **I** and **J**) β-catenin immunostaining. (G–I) Histology of a representative adenocarcinoma. Insets in A–F are magnified views of the rectangles. (H and J) Enlarged images of rectangles in G and I, respectively. The dotted line indicates the boundary between the β-catenin^IC^ and β-catenin^CM^ regions. Scale bars, 100 μm (A–F, H and J), 25 μm (insets) and 500 μm (G and I).

Histopathologically, around 70% of the small intestinal tumors of C3B6F1 *Apc^Min^*^/+^mice were adenomas without invasion, showing irregular glandular structures, increased nuclear-to-cytoplasmic ratio and cellular atypia compared with normal mucosa (Fig. 1A and B). Adenocarcinomas developed at lower frequencies and were characterized by their invasion into the submucosal layer (Fig. 1C), as reported previously [13]. To quantify the progression from adenoma to adenocarcinoma, tumors larger than 1.5 mm in diameter were randomly selected, sectioned, stained with H&E and analyzed histopathologically. Irradiation with γ-rays increased the proportion of adenocarcinoma in both male (33 vs 22% for nonirradiated, *P* < 0.05) and female mice (30 vs 16%, *P* = 0.06) (Table 2). Notably, tumor morphology did not differ between the nonirradiated and irradiated groups.

### The extent of intracellular accumulation of β-catenin varies within individual tumors

Previous studies using B6 *Apc^Min^*^/+^ mice revealed that β-catenin accumulates in both the nucleus and cytoplasm of tumors [21]. In C3B6F1 *Apc^Min^*^/+^ mice herein, β-catenin localized to the cell membrane (CM) [herein, named CM localization, β-catenin^CM^] of the normal intestinal epithelium (Fig. 1A and D) and accumulated in both the nucleus and cytoplasm [intracellular (IC) localization, β-catenin^IC^] of adenoma and adenocarcinoma (Fig. 1B, C, E and F), as previously reported [21]. Although histologically atypical cells composed the tumors, they were spatially heterogeneous in that the accumulation of β-catenin exhibited a mosaicism with certain regions showing substantial IC localization and other regions displaying mostly CM localization (Fig. 1G–J).

### The *Apc*^+^ allele is lost in β-catenin^IC^ regions within intestinal tumors

**Fig. 2:**
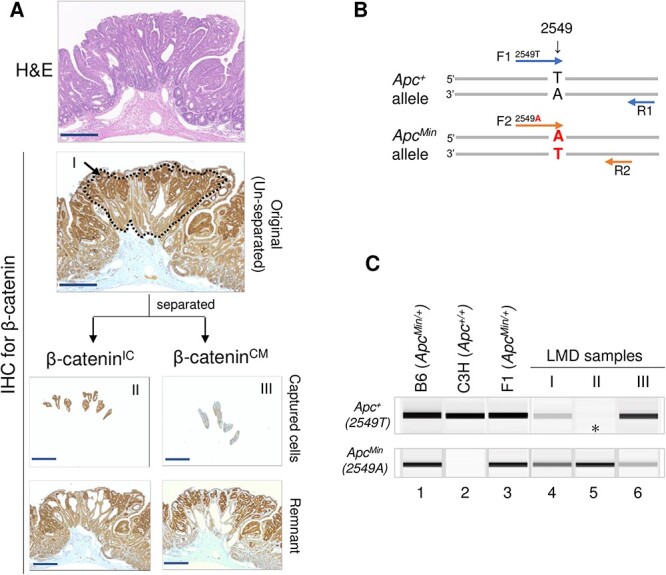
Immunoguided LMD approach for cell collection and analysis of *Apc*^+^ allele loss. (**A**) Guided by the nuclear accumulation of β-catenin, LMD was used to collect cells in the unseparated (I), β-catenin^IC^ (II) and β-catenin^CM^ (III) regions. IHC = immunohistochemical staining. (**B**) Primer set used to detect *Apc*^+^ and *Apc*^*Min*^ alleles. (**C**) LOH results showing loss of *Apc*^+^ in the β-catenin^IC^ region (asterisk). Lanes 1–3, ear-skin DNA from B6 *Apc^Min^*^/+^ , C3H *Apc*^+/+^ and C3B6F1 *Apc^Min^*^/+^ mice; lanes 4–6, samples corresponding to I, II and III in A. Results are representative of five tumors. Scale bars, 250 μm (A).

Previous analyses of intestinal tumors from hybrid (AKR × B6)F1 and (CBA × B6)F1 *Apc^Min^*^/+^ mice revealed a radiation-specific LOH pattern on chromosome 18, where *Apc* resides [14, 16]; these studies, however, used bulk tumor tissues without examining β-catenin expression. Our first analysis of LOH at the *Apc* locus did not give reproducible results using the bulk tumor samples from C3B6F1 *Apc^Min^*^/+^ mice. Because the IC accumulation of β-catenin is most likely due to the *Apc*^+^ allele loss, we hypothesized that the status of the *Apc*^+^ allele differs between the β-catenin^IC^ and β-catenin^CM^ regions. Therefore, we examined the presence of the *Apc*^*Min*^ and *Apc*^+^ alleles in the regions of intestinal tumors that differed with respect to β-catenin IC accumulation (Fig. 2). To isolate those regions, we first performed β-catenin immunostaining on formalin-fixed paraffin-embedded (FFPE) sections of tumors and used LMD to collect (i) whole-tumor tissues, (ii) β-catenin^IC^ regions and (iii) β-catenin^CM^ regions (Fig. 2A). DNA was extracted from each region and subjected to PCR with two forward primers differing in the specificity of the 3′ end (2549) of each of the *Apc^Min^* and *Apc*^+^ alleles to detect them independently (Fig. 2B and C). As expected, the two alleles were detected in ear-skin DNA of C3B6F1 *Apc^Min^*^/+^ (Fig. 2C, lane 3) and paternal B6 *Apc^Min^*^/+^ mice (Fig. 2C, lane 1), whereas only the *Apc*^+^ allele was detected in maternal C3H *Apc*^+/+^ mice (Fig. 2C, lane 2). Regarding the five tumor tissue samples isolated as illustrated in Fig. 2A, both the alleles were detected in the whole tumor (Fig. 2C, lane 4), whereas only the *Apc^Min^* allele was detected in the β-catenin^IC^ regions (Fig. 2C, lane 5) and both the alleles were detected in the β-catenin^CM^ regions (Fig. 2C, lane 6). These results suggested that tumorigenesis in the β-catenin^IC^ regions is initiated by the loss of the *Apc*^+^ allele and that the β-catenin^CM^ regions have distinct origins. Furthermore, an analysis of the whole tumor (i.e. including both the β-catenin^IC^ and β-catenin^CM^ regions, Fig. 2C, lane 4) revealed no evidence for the loss of the *Apc*^+^ allele, indicating that the combination of β-catenin immunostaining and LMD is necessary for accurate tissue collection.

### Choice of fixative and prestaining affect the subsequent analysis of paraffin-embedded samples

**Fig. 3:**
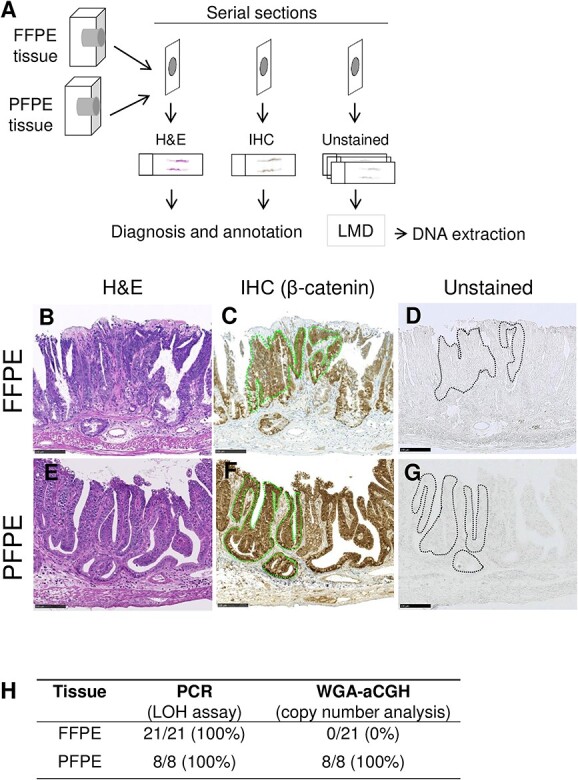
Effect of the formalin-free fixative PAXgene on tissue staining and DNA quality. (**A**) Staining and DNA extraction strategy using FFPE and PFPE samples. IHC = immunohistochemical staining. (**B**–**G**) Serial sections were used for diagnosis on H&E staining (B and E), β-catenin immunostaining (C and F) and DNA isolation without staining (D and G). Dotted lines in C, D, F and G identify the β-catenin^IC^ regions. (**H**) Summary of detection sensitivity of LOH and copy number variants at the *Apc* locus. Scale bars, 100 μm (B–G).

Because our results presented in Fig. 2 implied insensitivity of analyses using the whole tumor, we aimed to examine more precisely the status of chromosome 18 by a DNA copy number analysis combined with a LOH analysis in more homogeneous tumor segments in which we had defined the β-catenin expression in C3B6F1 *Apc^Min^*^/+^ mice. Technically, however, the quality of DNA recovered from the immunohistochemically stained FFPE tissue samples was not sufficient for comprehensive genomic analyses. Because formalin causes DNA crosslinks and thereby decreases the accuracy and reliability of subsequent DNA analyses, we substituted formalin with the PAXgene reagent, which is a formalin-free tissue fixative that preserves nucleic acids better than does formalin [21, 22] (Fig. 3A). Fortunately, the quality of the β-catenin staining in FFPE and PAXgene-fixed paraffin-embedded (PFPE) tissues was comparable (Fig. 3B–G). Because DNA recovery tends to be low in immunostained tissue samples [23], we took an approach in which β-catenin expression was identified by immunohistochemical staining (Fig. 3C and F) and DNA was recovered from the corresponding unstained samples of proximal tissue (Fig. 3D and G). With this approach, both FFPE and PFPE tissues yielded sufficient amounts of DNA for reliable and reproducible PCR analysis (Fig. 3H, ‘PCR’). With FFPE tissues, the copy number changes at the *Apc* locus were not detectable using WGA and oligonucleotide aCGH technologies; with PFPE tissues, however, a decrease in copy number was indeed detected (Fig. 3H, ‘WGA-aCGH’).

### Pattern of chromosome 18 LOH in β-catenin^IC^ regions correlates with etiology

**Fig. 4:**
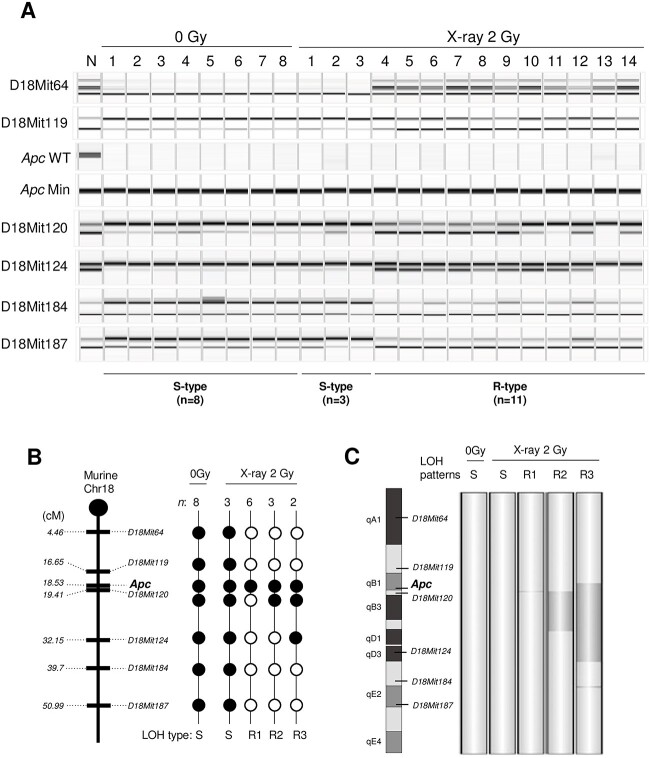
LOH and aCGH analyses using PFPE intestinal tumor samples. (**A**) LOH analysis of six microsatellite markers and the *Apc* locus on chromosome 18 in LMD samples from the 0 Gy (*n* = 8) and 2 Gy X-ray exposure groups (*n* = 14). N = normal tissue. (**B**) Mapping of the LOH results. Solid circle, LOH; open circle, no LOH. *n*, number of cases with the indicated LOH pattern; S = spontaneous type; R = radiation-induced types (R1–R3). (**C**) Changes in DNA copy number identified by aCGH analysis. Dark gray denotes regions with copy number loss detected by aCGH.

The LOH of six microsatellite markers on chromosome 18 was analyzed using DNA obtained from normal ear-skin samples and intestinal tumors (nonirradiated, *n* = 8; 2-Gy X-irradiated, *n* = 14). For the intestinal tumor samples, tissues of the β-catenin^IC^ regions were collected from unstained PFPE samples. LOH at the *Apc* locus was observed in all tumors from the nonirradiated (8 of 8) and irradiated (14 of 14) groups (Fig. 4A and B). The pattern of LOH was dichotomous, including those spanning nearly the entire chromosome and those beginning upstream of *Apc* and ending in the middle of the chromosome (Fig. 4B). Interestingly, the latter pattern was observed only in the irradiated group (11 of 14, 79%), whereas the former pattern was observed in both nonirradiated (8 of 8, 100%) and irradiated (3 of 14, 21%) groups. These results indicated that the latter and former LOH patterns were characteristics of the radiation-induced and spontaneous tumors, and thus we refer to them as ‘R’ and ‘S’ types, respectively (Fig. 4). In addition, the LOH analysis of tumor tissues exhibiting β-catenin accumulation provided the information for accurate prediction of etiology of the intestinal carcinogenesis.

Whether copy number losses associate with such LOH in radiation-induced tumors has not been addressed in previous studies on *Apc^Min^*^/+^ mice [14–16]. To evaluate the pattern of DNA copy number aberrations, tumors displaying representative LOH patterns were chosen from each group for aCGH analysis (i.e. 1 with S-type LOH in the nonirradiated group, 1 with S-type LOH in the irradiated group and 3 with R-type LOH in the irradiated group). Tumors with the S-type LOH pattern exhibited normal copy numbers throughout chromosome 18, indicating that the LOH was caused by chromosomal mis-segregation or mitotic recombination (Fig. 4C). In contrast, a tumor with the R-type LOH pattern harbored copy number losses spanning chromosome 18qB1–D3 (Fig. 4C), indicating that this type of LOH was caused by interstitial deletion. These results suggest that the interstitial (or R-type) LOH pattern is most likely caused by an interstitial chromosomal deletion and hence is a signature for radiation-associated tumors.

## DISCUSSION

In this study, we characterized radiation-induced intestinal tumors, using C3B6F1 *Apc^Min^*^/+^ mice. Our results clarify the mosaicism of β-catenin overexpression in intestinal tumors of *Apc^Min^*^/+^ mice and identify novel differences in genomic variation (presence or absence of the *Apc*^+^ allele) indicative of tissue mosaicism, i.e. loss of the *Apc*^+^ allele in the β-catenin^IC^ region and retention of the *Apc*^+^ allele in the β-catenin^CM^ region. In addition, by devising a sample recovery method, we were also able to show for the first time in F1 *Apc^Min^*^/+^mice that deletion of the *Apc* site predicted by LOH analysis was confirmed by aCGH analysis. Our study establishes a highly sensitive method for classifying dichotomous LOH types, namely chromosomal mis-segregation/mitotic recombination (S type) and interstitial deletion (R type), and demonstrates that the R-type LOH is a signature of radiation-induced intestinal tumors.

Herein, β-catenin expression was mosaic in the intestinal tumors of C3B6F1 *Apc^Min^*^/+^ mice (Fig. 1). The mosaicism of β-catenin has been reported in human colorectal tumors [24, 25]. The mechanism underlying this heterogeneity is not well understood, although intratumor heterogeneity is sometimes related to genetic [26] rather than epigenetic changes [27]. This heterogeneity has been reported for intestinal tumors of *Apc^Min/^*^+^ mice [28, 29] as well as patients of FAP [30, 31], implying that distinct polyp types from different mammals may have a similar physiological basis. Although previous studies have used F1 *Apc^Min^*^/+^ mice to suggest radiation-specific LOH patterns in bulk intestinal tumors [14, 16], those studies identified a subset of tumors displaying retention of the *Apc*^+^ allele, implying substantial contamination of normal cells or heterogeneity of the tumor tissue. In addition, these studies never associated LOH with copy number variation. A major challenge has been the difficulty in obtaining homogenous tumor samples with a sufficient amount and quality of DNA for aCGH experiments. In this study, the immunoguided LMD method was used to collect tumor samples based on the tissue phenotype instead of collecting bulk tumors. Specifically, we established an accurate LOH determination method by collecting and analyzing tumor cells with a common tumorigenesis mechanism using β-catenin accumulation as an indicator, in accordance with the molecular mechanism of multistep tumorigenesis triggered by *Apc* gene inactivation. We additionally overcome the abovementioned challenge by combining the use of a formalin-free fixative, an LMD approach guided by immunostaining images of proximal sections, and the application of WGA before aCGH. Haines *et al*. [15] previously collected tumor tissues for LOH analysis using pathological (i.e. H&E-stained) images as a guide. The application of LMD to immunostained tissues has been shown effective in separating cells that are morphologically very similar but have immunohistochemically discernible characteristics [32], although the integrity of the analytes (e.g. DNA) in the processed samples is often compromised by the use of a formalin-containing fixative and the staining procedure itself [23]. We herein combined those techniques to confirm that all tumors of *Apc^Min^*^/+^ mice lacked the *Apc*^+^ allele, in contrast to what has been reported in studies using bulk tumors [14, 16]. For the first time, we further associated these LOH patterns with copy number variations. We thus could identify chromosomal mis-segregation/mitotic recombination (S type, i.e. LOH with two copies) and interstitial deletion (R type, i.e. LOH with one copy) events that affect the *Apc*^+^ allele, with the latter occurring only in the irradiated group, thus corroborating and extending the results reported for F1 *Apc^Min^*^/+^ mice [14–16]. The method adopted herein offers a promising tool for identifying radiation-induced oncogenic changes in other animal models.

In the irradiated group, we identified not only tumors with the R-type LOH pattern (R-type tumors) but also those with the S-type LOH pattern (S-type tumors) (Fig. 4). This is consistent with the results obtained with *Ptch1*^+/−^ mice and *Tsc2*^+/−^ Eker rats [19, 20, 33, 34], each of which is also a model animal with germline heterozygous deletions of a tumor suppressor gene. Therein, the remaining wild-type allele undergoes spontaneous deletion, leading to the development of S-type tumors in nonirradiated animals, whereas both S- and R-type tumors develop in irradiated animals. Especially in *Ptch1*^+/−^ mice, which develop only one tumor per individual, the incidence of R-type tumors increases, whereas that of S-type tumors decreases in a dose-dependent manner [33]. Therefore, it is possible that competition between cells with S-type and R-type LOH patterns would result in a reduced incidence of S-type tumors. The possibility remains, however, that radiation-induced changes in the intestinal microenvironment may increase the incidence of S-type tumors. To test this possibility, it will be meaningful to analyze the dose dependence of the incidence of S- and R-type tumors in F1 *Apc^Min^*^/+^ mice, which develop multiple intestinal tumors in each individual mouse.

Studies of radiation carcinogenesis in rodent models have shown that chromosomal deletions affect tumor suppressor genes in mouse acute myeloid leukemia [35], lymphoma, sarcoma and mammary carcinoma in *Trp53*^+/−^ mice [36–38], intestinal tumors in *Apc*-deficient mice [14–16, 39], medulloblastoma in *Ptch1*^+/−^ mice [33, 40], renal tumors in *Tsc2*^+/−^ Eker rats [34] and rat mammary cancer [41–44]. This is a feature not often seen in tumors of nonirradiated animals and is most likely attributable to radiation exposure. *Apc^Min^*^/+^ mice, *Ptch1*^+/−^ mice and *Tsc2*^+/−^ Eker rats are special animal models of carcinogenesis owing to a heterozygous germline mutation of a cancer driver gene, and thus it must be considered whether LOH and copy number variations that are a consequence of chromosomal structural instability caused by the absence of one allele can be a general mechanism. To date, only medulloblastoma in *Ptch1*^+/−^ mice and renal tumors in *Tsc2*^+/−^ Eker rats have been properly demonstrated (i.e. with evidence of copy number losses) to have an interstitial deletion as a radiation signature in solid tumors [19, 20, 33, 34]. Because these tumors are not of much relevance to human radiation risk, it is significant that we extended these findings to a model of intestinal tumors, one of the diseases relevant to human radiation risk. Furthermore, even in wild-type mice, deletion of any one of the tumor suppressor genes *Pax5*, *Sfpi1*, *Ikzf1*, *Pten* or *Cdkn2a* has been reported specifically in irradiated animals in copy number analyses of lymphomas, acute myeloid leukemia and thymic lymphoma [35, 45, 46]. Thus, we speculate that the inactivation of tumor suppressor genes via interstitial deletion is a common mechanism of radiation-induced carcinogenesis. This mechanism could be used for discriminating radiation-induced tumors of *Apc^Min^*^/+^ mice exposed to low levels of radiation from those developed by spontaneous mechanisms, as previously performed with *Ptch1*^+/−^ mice [19, 20].

An important unresolved issue in radiation protection is whether there is a dose-dependent increase or threshold dose of cancer risk in the dose range of several tens of mGy. We anticipate that this approach will help clarify the effects of low dose and low dose rate radiation.

## Data Availability

The microarray data are available in the Gene Expression Omnibus database under accession number GSE228693.
